# Effect of Mannoprotein-Producing Yeast on Viscosity and Mouthfeel of Red Wine

**DOI:** 10.3390/foods14030462

**Published:** 2025-02-01

**Authors:** Emerson Núñez, Josefina Vidal, Matías Chávez, Edmundo Bordeu, Fernando Osorio, Sebastián Vargas, Elba Hormazábal, Natalia Brossard

**Affiliations:** 1 Department of Fruit Production and Enology, School of Agricultural Science and Natural Systems, Pontificia Universidad Católica de Chile, Av. Vicuña Mackenna 4860, Macul 7820436, Chile; ennunez@uc.cl (E.N.); jvvidal1@uc.cl (J.V.); mjchavez@uc.cl (M.C.); ebordeu@uc.cl (E.B.); 2 Department of Food Science and Technology, Technological Faculty, Universidad de Santiago de Chile, Av. El Belloto 3735, Estación Central, Santiago 9170125, Chile; fernando.osorio@usach.cl; 3 Center for Research & Innovation, Viña Concha y Toro, Pencahue 3550000, Chile; sebastian.vargass@conchaytoro.cl; 4 Almaviva Vineyard, Viña Concha y Toro, Pencahue 8166174, Chile; ehormazabal@almaviva.cl

**Keywords:** mannoproteins, yeast, mouthfeel, viscosity, sensory correlation

## Abstract

Mannoproteins enhance wine stability and sensory properties, but their specific role in modulating viscosity and mouthfeel across wine quality levels remains underexplored. This study explores the nuanced impact of various mannoprotein-producing yeast strains on specific mouthfeel sensations, particularly emphasizing viscosity, across Standard and Premium quality tiers of Cabernet Sauvignon commercial wines. The aim was to understand the intricate relationship between yeast-derived mannoproteins and the broader sensory landscape of red wines. The methodology encompasses a comprehensive mannose extraction method, rheological measurements, and sensory Rate-All-That-Apply evaluations, all of which are integrated into a Principal Component Analysis. The results showed slight color variations due to the wine spending one month on lees. A positive correlation was found between mannose content and viscosity in only Standard-quality wines. The correlation with sensory data indicated a strong relationship between volume, viscosity, and mannose content in Premium-quality wines, which was less pronounced in Standard-quality wines. Furthermore, parameters related to mouthfeel quality, such as roundness and smoothness, were also associated with these findings. Prospects involve further exploration of correlations between mouthfeel sensations, sensory descriptors, and the structural characteristics of mannoproteins, aiming for a more comprehensive understanding of the intricate interplay in wine composition.

## 1. Introduction

Mannoproteins are yeast cell wall components released from yeast autolysis during or after alcoholic fermentation and aging on lees [1,2]. Mannoproteins impart positive effects on the technological properties of wines, constituted primarily by mannose (85–90%) and proteins (10–15%). These effects include color and colloidal stabilization, leading to greater visual clarity in the final product, reduction in tannin–protein aggregation, and improvement in the in-mouth characteristics of red wines, such as rheological properties [1,3,4]. Specifically, these molecules can influence the mouthfeel perception of the “body” by increasing the sensation of viscosity [5]. Proposed mechanisms are based on the interaction of mannoproteins, forming stable complexes between H-bonds and major solvents in wines (H_2_O, Ethanol), leading to a decrease in these free molecules and an increase in wine viscosity [2,6].

Commercial products rich in mannoproteins can be added, but the alternative of using mannoprotein-producing yeast can represent a significant advantage, even from an economic standpoint [7]. Mannoproteins find applications in various wines, including white, red, rosé, and white sparkling wines. In commercial winemaking, the most frequent practice for enhancing mouthfeel and stability involves adding exogenous mannoprotein-rich products during the final stages of wine production. These additions are primarily applied to white and sparkling wines to improve protein stability, clarity, and smoothness. In contrast, mannoprotein applications are rarely used in red wines, particularly those rich in astringent and bitter tannins resulting from prolonged maceration [8,9,10]. The relationship between mannoproteins and sensory perception varies depending on the wine matrix; in white wines, mannoproteins enhance smoothness and reduce protein haze, while in red wines, they interact with polyphenols to soften astringency and increase viscosity [11,12]. However, the direct contribution of mannoprotein-producing yeasts to sensory attributes during fermentation has been less explored, especially across different wine quality levels, making this study a novel contribution to understanding how yeast selection influences mouthfeel and viscosity. On the other hand, the contact time between mannoproteins and red wine is crucial to achieve the colloidal state capable of inducing a significant variation in perceived sensations [9].

Wines create mouthfeel sensations of astringency, body, balance, and viscosity [12,13]. These non-taste sensations are produced by oral tactile stimulations, which are not fully understood at present. Different names have been used to characterize them, with the term “mouthfeel” being more commonly used to refer to sensations related to tactile responses in the mouth [14]. Wine mouthfeel can be characterized by a combination of instrumental techniques based on fluid behavior, but limitations exist as wine sensations go beyond flow. Viscosity is probably one of the best properties that is correlated with wine body since it affects the thickness sensation in the mouth [15]. Other instrumental techniques are also needed to complete the characterization of mouthfeel. Nonetheless, mannoproteins play a significant role in contributing fullness and smoothness sensations to wine [16]. Certain physical parameters, including viscosity and the friction coefficient, may provide information enabling scientists to correlate physical quantities with numerous sensory perceptions [17].

In the context of this investigation, our principal objective was to conduct a comprehensive assessment of how diverse mannoprotein-producing yeasts exert influence on specific mouthfeel sensations, with a specific emphasis on viscosity, in red wines spanning various quality levels, ranging from Standard to Premium grades. This analysis is designed to provide a nuanced comprehension of the intricate interplay between yeast-derived mannoproteins and the sensory attributes contributing to the wine-tasting experience. Going beyond a mere examination of the impact of mannoproteins on viscosity, this study aspires to delve into a broader sensory landscape by exploring the correlations between mouthfeel sensations and their respective sensory descriptors. Through the elucidation of these connections, we aim to provide valuable insights for winemakers, establishing a foundation for well-informed decisions in the utilization of mannoproteins to enhance and differentiate wines across quality categories.

## 2. Materials and Methods

### 2.1. Red Wines and Yeast Samples

The analysis was carried out at the Agricultural and Oenological Laboratory of the Concha y Toro Innovation and Research Center and the Oenological Laboratory of the School of Agricultural and Natural Systems at the Pontificia Universidad Católica de Chile. Six Cabernet Sauvignon wines were analyzed. These wines were produced with six different mannoprotein-producing yeasts, a control (Y1) and five yeast selections with high mannoprotein production (Y2, Y3, Y4, Y5, and Y6). The control wine was fermented using a commercial yeast strain with standard mannoprotein production, serving as a baseline for comparison with high-mannoprotein-producing yeast strains. While mannoprotein concentrations in grape juice were not explicitly measured before fermentation, the experimental design considered wines produced under controlled fermentation conditions, ensuring consistent comparisons. Wines were made from Standard (S)- and Premium (P)-quality grapes (six wines for each quality) with three replications per wine coming from the industrial scale at Concha y Toro vineyards. All measurements were performed in triplicate on three bottles of wine from three vats. These bottles were independently produced from three different fermentation tanks, ensuring the replicates represented true biological variability rather than pseudo-replication. This approach allows us to capture variations that arise from the winemaking process while minimizing technical variability. The fruit used came from 6 different Maule vineyards with similar growing conditions: Lourdes, Palo Santo, El Boldo, Idahue, Quebrada de Agua, and Requínoa.

### 2.2. Winemaking

Grapes were manually harvested, transported, received, and weighed upon arrival (approximately 850 kg). Harvested bunches were destemmed and crushed using a Delta de-stemmer (Bucher^®^, Frutigen, Switzerland) and then transferred to the fermentation bins (1220 × 1220 × 770 mm, Polypropylene [PP], 857 L capacity). The fermentation process began with a 5 min punching down to homogenize the mixture. Then, samples were taken for analysis using a Y15 automatic analyzer (Biosystem^®^, Barcelona, Spain) (Soluble Solids, SS [°Brix], titratable acidity, TA [g tartaric acid L^−1^], pH, Sulfur Dioxide, SO_2_ [mg SO_2TOTAL_ L^−1^], Malic Acid [gL^−1^], Yeast Assimilable Nitrogen [YAN]). Afterward, the mixture was homogenized again through a 5 min punching down. Finally, 6 different yeasts, previously rehydrated, were applied at 20 g hL^−1^, and the temperature during fermentation was maintained at around 26 °C. When the density reached 1060 [kg/m^3^], O_2_ (8 mg L^−1^) and diammonium phosphate (DAP, 20 g hL^−1^) were applied. Several punch-downs were performed during fermentation: two 5 min punch-downs per day until 1060 [kg/m^3^], two 2 min punch-downs per day until 1020 [kg/m^3^], and, finally, for densities below 1020 [kg/m^3^], punch-downs were continued twice daily for approximately 1 min each to submerge the cap efficiently. Density and temperature were monitored daily, four times a day. Racking was carried out on the 5th day of alcoholic fermentation (AF), retaining only the drained wine. For malolactic fermentation (MLF), the wine was transferred to a 100 L tank when sugars were below 2 gL^−1^. pH was adjusted to 3.5 and wines were inoculated with *Lactic Viniflora^®^* CH16 Hansen bacteria (4 g hL^−1^). The tank was kept in a heated chamber at temperatures between 20 and 22 °C. Laboratory samples were retaken for analysis using the Y15 equipment once a week to monitor malolactic fermentation and volatile acidity. Once MLF was complete, the wine was racked, treated with 4 g hL^−1^ of sulfur dioxide, and homogenized with N_2_, and the pH was adjusted to 3.5, while SO_2_ was adjusted to 35 mgL^−1^. The wine was kept in tanks with its fine lees without stirring for one month, then bottled with SO_2_ adjusted to 35 mgL^−1^, filtered through 5–3 and 1 um plate membranes, and finally stored at 12 °C.

#### Red Wine Characterization

Wines were classified as Standard or Premium based on the grape quality (blend vs. premium) and ripening stage at harvest. Premium wines were produced from high-quality grapes with more favorable ripening conditions, resulting in higher phenolic content and sensory complexity. Wine was characterized based on its alcohol content (*v*/*v*), titratable acidity (g tartaric acid L^−1^), pH, -glucose + fructose (gL^−1^), and acetic acid (g acetic acid L^−1^). In addition, absorbance measurements at OD 420 nm, OD 520 nm, and OD 620 nm were taken to obtain color intensity and hue using a UV-VIS Orion Aquamate 8000 spectrophotometer (Thermo Scientific^®^, Waltham, MA, USA), ensuring a clear distinction between the two quality levels. All analyses were performed in triplicate.

### 2.3. Mannose Determination

The method was carried out based on the study of Guadalupe et al. (2010) [18]. Mannoproteins are mainly found in the outermost layer of the yeast cell wall, and they are composed of 30% protein fraction and 70% sugars, of which 98% are mannose [19]. There are different methods to concentrate and measure it. Our analytical proposal is based on the ultrafiltration method using centrifugal filtration tubes. This method of measuring mannoprotein consists of concentrating the wine sample by ultra concentration with Amicon^®^ centrifugal filters, and subsequent measurement of mannose, post hydrolysis, using an enzymatic method. The wine was centrifugated at 3220× *g* for 20 min at 4 °C (Eppendorf^®^ Centrifuge 5804R A-4-81 Rotor, Hamburg, Germany). Colloidal polysaccharides were first precipitated in Falcon tubes with 100 mL of 95% ethanol and 20 mL of the sample (5 volumes of ethanol) for 12 h at 4 °C. The sample was then centrifuged at 9800× *g* for 20 min (Eppendorf^®^ Centrifuge 5804R, F-34-6-38 Rotor, Hamburg, Germany). The pellets obtained were washed twice with 10 mL of 95% ethanol and centrifuged for 10 min in each wash. To concentrate the 20 mL samples, ultrafiltration was performed using centrifuge tubes (Merk Amicon^®^ 10 KDa, Darmstadt, Germany). Each 20 mL sample was concentrated 80 to 100 times using Amicon^®^ 10 kDa tubes, and centrifuged at 3000× *g* at 25 °C for 20 min. Since the tube capacity was 15 mL, this centrifugation was performed twice. The pellet obtained from the concentration step was resuspended with 5 mL of deionized water in sealed 30 mL glass tubes (PTFE Screw Cap Cultures 18 × 180 mm; Sigma-Aldrich^®^, San Louis, MO, USA). It was carefully extracted to resuspend and transfer the concentrate, and 1 mL of deionized water was added. The tube was then sealed and agitated using a vortex mixer. This operation was repeated until approximately 5 mL of the sample was obtained. For hot acid hydrolysis of polysaccharides, 415 μL of 96% H_2_SO_4_ was added. After sealing, the tubes were agitated on a vortex mixer, then placed in an oven (Binder^®^ FD 260, Tuttlingen, Germany) at 100 °C for 180 min, and then cooled to room temperature.

After hydrolysis, the 5 mL samples were transferred to 50 mL Falcon tubes, with rinsing being performed several times with 5 mL of deionized water (MiliQ; Merck Millipore^®^, Burlington, MA, USA) to transfer the entire hydrolysate to the Falcon tube. The samples were neutralized to pH ≈ 7.0 using NaOH with solutions of 5, 3, 2, 1, and 0.1 M, sequentially used to control the solution’s pH without exceeding a sample volume of 20 mL. This process was conducted with the assistance of a pH meter, and manual agitation was performed throughout. Due to the presence of insoluble particles after hydrolysis and neutralization, the samples were centrifuged again at 9800× *g* for 30 min to remove the insoluble particles. Finally, to reach an estimated concentration ranging from 40 to 800 mgL^−1^ of mannose for enzymatic determination, the sample was quantitatively transferred to 20 mL volumetric flasks and filled with deionized water. This was performed considering that wines have an approximate mannoprotein concentration of 100–150 mgL^−1^. The amount of mannose was measured using the D-Mannose, D-Fructose, and D-Glucose enzymatic kit (Megazyme Ltd., Bray, Ireland). Two replicates were performed for each wine sample analyzed.

### 2.4. Rheology

To carry out the experiments, a rheometer (Discovery Hybrid Rheometer HR2, Software TRIOS v3.3.0.4055, New Castle, DE, USA) with a cone and plate configuration was used (cone angle 1.008°; diameter 60 mm; 27 μm gap between cone and plate). The lower plate was equipped with a Peltier temperature control system. The wine samples were deposited on the lower plate. The temperature was maintained at 28 °C to mimic the temperature of the oral cavity [20]. To ensure that the samples reached and maintained the working temperature, a stabilization period of 5 min was applied, as provided by the instrument’s software. For the flow behavior of the samples, data were obtained at a shear rate ranging from 0.1 to 300 s^−1^ (ascending). The flow curve data were fitted to the linear model as follows:

(1) $$\sigma =m\cdot \dot{\gamma }$$

where σ is the shear stress (Pa), m is the slope of the line represented by viscosity (Pa s), and $\dot{\gamma }$ is the shear rate (s^−1^). All measurements were performed in triplicate.

### 2.5. Sensory Analysis

The evaluation of the samples was conducted by separating the wine qualities into Standard and Premium categories. Twenty-four panelists evaluated the six Standard wines, while twenty panelists assessed the Premium wines. All sensory evaluations were performed in person at the Center for Research and Innovation of Viña Concha y Toro under controlled conditions, adhering to ISO Standard Methods (e.g., ISO 8586) [21]. The evaluation followed the Rate-All-That-Apply (RATA) methodology, a variation of the commonly used Check-All-That-Apply (CATA) question format [22]. This sensory tool allows for detailed product characterization and enhances product discrimination through the use of intensity scales [23]. A structured intensity scale from 1 to 5 was employed to evaluate nine sensory attributes: volume, fat, velvety astringency, drying astringency, bitterness, smoothness, structured, roundness, and quality in the mouth. These attributes were collaboratively selected by Viña Concha y Toro professionals based on the existing literature [12,24]. The panelists were all expert oenologists from Viña Concha y Toro, each with more than 10 years of experience in sensory evaluation, with an age range of 35 to 55 years and balanced gender representation. Wine samples were stored in cellars at a controlled temperature of 15 °C with minimal light exposure to preserve their integrity. On the day of the evaluation, the wines were climatized in a sensory evaluation room maintained at 18 °C. Samples were presented in ISO-standard wine-tasting glasses to minimize external variability. Panelists assessed the samples individually and recorded their evaluations via an online platform, which was used solely for data recording. The samples were randomly presented to each panelist, and the attributes of each wine were also randomized on the evaluation form. Panelists were allowed to fill out the form only once, without the option to revisit or modify their entries. Water was provided for palate cleansing throughout the tasting session.

### 2.6. Statistical Analysis

Statistical analysis was carried out using STATGRAPHICS Centurion XVI, v.16.1.03 (Statgraphics Technologies, The Plains, VA, USA), and Principal Component Analysis (PCA) was performed using XLSTAT software v.2024.4.0 (1424) (Addinsoft, Paris, France). Analysis of Variance (ANOVA) was performed to evaluate the effect of yeasts, and Tukey’s means comparison was used to determine significant differences between treatments. PCA was used to describe the relation between mannose concentration, viscosity, and sensory results. The significance level used was *p* < 0.05 throughout the study. All analyses were performed in triplicate.

## 3. Results

### 3.1. Chemical Characterization

Table 1 displays the chemical composition of wines fermented with different mannoprotein-producing yeasts in Standard (S)-quality Cabernet Sauvignon wines. These parameters include alcohol content, titratable acidity, acetic acid, pH, residual glucose, fructose, color intensity, and hue. While no significant differences were observed in alcohol content or color intensity among the strains (*p* > 0.05), significant differences were detected in titratable acidity, acetic acid, pH, and residual glucose/fructose (*p* < 0.05), suggesting that the yeast strain used may influence certain chemical and organoleptic characteristics of the wine.

Table 2 displays the chemical composition of Premium (P)-quality Cabernet Sauvignon wines fermented with different mannoprotein-producing yeasts. In this quality of wine, no significant differences were found in alcohol content, pH, residual sugars, or hue among strains (*p* > 0.05). However, significant variations were observed in titratable acidity, acetic acid, and color intensity (*p* < 0.05).

### 3.2. Mannose Content

Table 3 shows the analyses of the mannoprotein concentrations in Cabernet Sauvignon wines fermented with different mannoprotein-producing yeast strains, categorized into Standard and Premium quality levels. While most comparisons between Standard- and Premium-quality wines within each strain showed no significant differences (*p* > 0.05), strain Y3 demonstrated a notable reduction in mannose concentration in Premium wines compared to Standard wines (*p* = 0.0331). Furthermore, significant differences in mannose concentrations were observed among yeast strains in Standard-quality wines (*p* = 0.0341); however, all strains can be considered as mannoprotein overproducers.

### 3.3. Rheological Behavior

Table 4 presents an analysis of the rheological behavior of Cabernet Sauvignon wines influenced by different mannoprotein-producing yeast strains, distinguishing between Standard and Premium quality categories. Minor variations in viscosity were observed within each quality level; however, when comparing quality categories, Premium wines exhibited notably higher viscosity than Standard wines.

**Figure 1:**
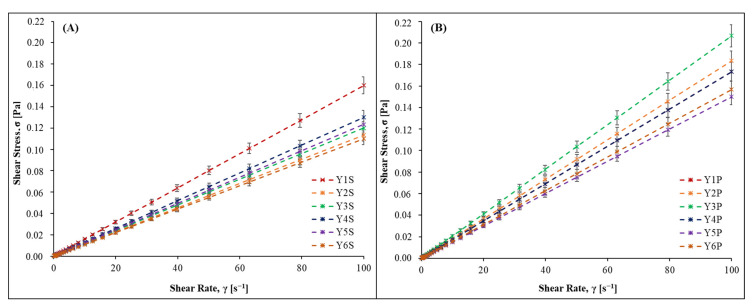
Flow behavior for mannoprotein-producing yeasts in (**A**) Standard (S) and (**B**) Premium (P) Cabernet Sauvignon wines at a shear rate between 0.1 and 100 s^−1^.

Figure 1 represents the rheological behavior of wines treated with different mannoprotein-producing yeasts for both qualities of red wines at an ascending shear rate from 0.1 to 100 s^−1^. Here, we can see that the control yeast had a higher viscosity in Standard-quality wines; however, in Premium-quality wines, the values were higher for all strains. All of them presented a typical Newtonian behavior for this type of fluid.

**Figure 2:**
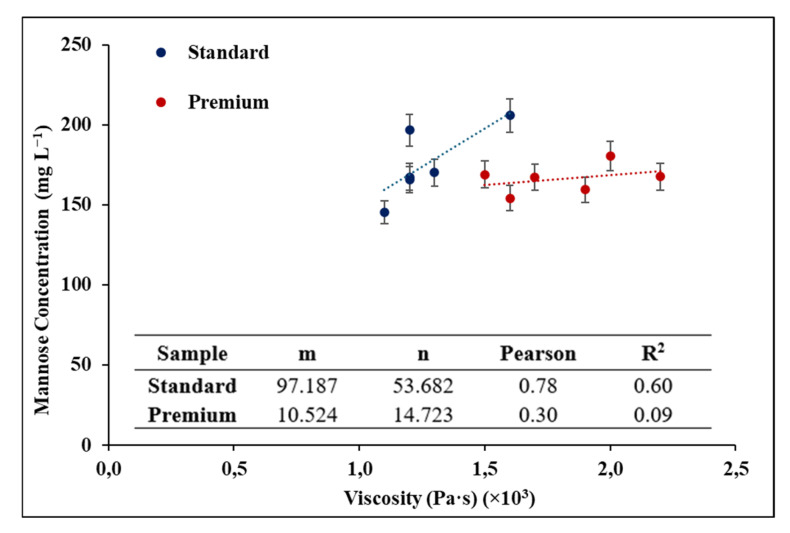
Correlation between viscosity and mannoprotein content for different yeasts and quality of red wines.

Figure 2 shows the relationship between viscosity and the concentration of mannoproteins for each yeast strain by wine quality where a better correlation is shown in Standard-quality wines.

### 3.4. Sensorial Attributes

The sensory attributes of Standard- and Premium-quality Cabernet Sauvignon wines were significantly influenced by the different mannoprotein-producing yeast strains, as indicated by the Rate-All-That-Apply (RATA) analysis presented in Table 5 and Table 6.

In general, both cases exhibited similar variations, with Y1 being the yeast strain that most significantly enhanced the quality parameters of the wines. This strain showed strong correlations with physical parameters, such as viscosity, and chemical parameters, including each wine’s profile and mannose content.

**Figure 3:**
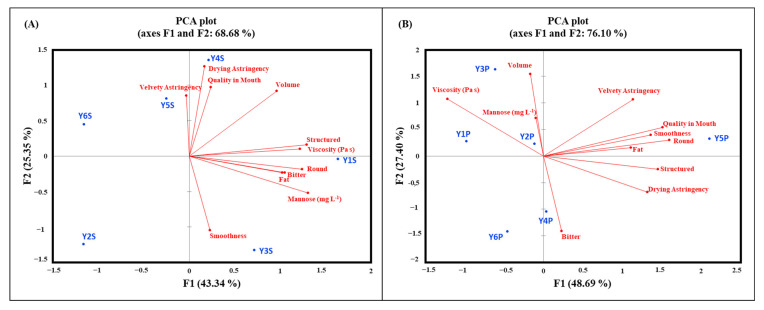
Principal Component Analysis of the mannoprotein-producing yeasts in (**A**) Standard (S) and (**B**) Premium (P) Cabernet Sauvignon wines, including the sensory data, the mannose concentration, and viscosity.

Figure 3 illustrates the correlations between sensory and physicochemical parameters—specifically, mannose concentration and viscosity—within a Principal Component Analysis (PCA) plot, while Table 7 provides detailed findings from the PCA conducted on sensory attributes, mannose concentration, and viscosity in Cabernet Sauvignon wines.

In both cases, direct relationships between mannose concentration and viscosity were evident, along with associated sensory attributes such as body, smoothness, and volume. These factors provide a substantial explanation for the observed correlations among the measured parameters. The relationships presented in Table 7 reveal notable differences between Standard- and Premium-quality wines regarding the correlation between mannoprotein concentration, viscosity, and sensory attributes. In Standard wines, a strong positive correlation was observed between mannose concentration and viscosity (F1: 0.921 and 0.857, respectively), indicating that the yeast strains, particularly Y1, contributed to enhanced viscosity through higher mannoprotein production. This trend aligns with previous studies highlighting the role of mannoproteins in increasing perceived mouthfeel viscosity by modulating the wine’s colloidal structure [9]. Wines fermented with Y1 demonstrated a pronounced association with sensory attributes such as roundness, volume, and structure, emphasizing the yeast’s contribution to improving overall tactile sensations.

Conversely, in Premium-quality wines, the correlation between mannose concentration and viscosity was less pronounced (F1: −0.058 for mannose and −0.736 for viscosity), suggesting that additional factors, such as ethanol concentration, phenolic compounds, or polysaccharide–tannin interactions, may play a more significant role in determining viscosity and associated mouthfeel attributes. This highlights the complexity of Premium wines, where mannoproteins alone may not fully explain the sensory profiles observed. Interestingly, yeast Y5 demonstrated strong contributions to smoothness, velvety astringency, and roundness, suggesting a synergistic effect between mannoproteins and the structural components of high-quality wines [18].

## 4. Discussion

### 4.1. Chemical Characterization

In Standard-quality wine, the alcohol content (*v*/*v*) exhibited no significant differences (*p* < 0.05), probably because other factors, such as initial sugar content and fermentation conditions, played a more dominant role in determining the final alcohol concentration [16]. In contrast, significant differences were observed in titratable acidity (g L^−1^) and pH. Yeast strains Y2 and Y4 seemed to increase the acidity of the wines. However, a lower pH in those treatments was not observed, except in the case of Y3 [25,26]. Acetic acid (g L^−1^) levels were all low, but strains Y1, Y3, and Y4 were slightly higher.

Final sugar concentrations (-glucose + fructose g L^−1^) were all low, corresponding to complete fermentations, with Y6 being particularly low. However, there was no impact on taste or microbiological stability [27]. No significant differences were detected in color intensity and hue, even though mannoproteins are supposed to contribute to color stabilization [28]. All these findings together reflect a modest influence of yeast selection as an active contributor to the basic structure of wines [29].

The analysis of Premium-quality Cabernet Sauvignon wines, fermented with various mannoprotein-producing yeast, reveals significant variations in some parameters (Table 2). Parameters such as titratable acidity, acetic acid, pH, and color intensity exhibited significant differences (*p* < 0.05). The influence of yeast selection on titratable acidity was also significant, but, in this case, Y1 increased acidity. pH was not affected significantly. Volatile acidity was, again, low, but Y1 and Y3 also had slightly higher levels, as with Standard-quality wines [30].

While certain parameters exhibited significant differences in Standard-quality wines, their variations were less pronounced or absent in Premium-quality wines. This suggests that the chemical attributes of Premium-quality Cabernet Sauvignon wines may be less sensitive to yeast-induced variations in certain parameters. An interesting difference in color characteristics was significant, with Y2 imparting the highest color intensity among Premium-quality wines. This has been attributed to the stabilizing effect of mannoproteins on anthocyanins and, in particular, colloidal color [31]. This difference may be particularly valuable for winemakers seeking to emphasize the color characteristics of Premium-quality Cabernet Sauvignon wines.

Color differences were observed between Premium-quality wines and Standard wines, with the former presenting a greater intensity; however, they also presented differences between them (*p* < 0.05). It has been reported that the addition of yeasts that produce mannoproteins can contribute as a colloidal stabilizer of wine pigments [32], which could explain the stability in the Premium-quality wines; however, the short time on the lees was not enough to visualize a better effect.

### 4.2. Mannose Concentration

Mannoproteins, characterized as polysaccharides, contribute significantly to sensory attributes [33]. In Standard-quality wines, the data unveiled some differences in mannose concentration among different yeast treatments (*p* < 0.05). Of particular significance was the higher mannose concentration observed in Y1-treated wines, indicating its potential to enhance the mannoprotein content in this specific wine category [34]. The higher mannose concentration associated with Y1 could be attributed to the yeast’s metabolic activities, which potentially favors the production or retention of mannoproteins [35,36]. No differences were found among the other strains.

Conversely, in Premium-quality wines, the differences in mannose concentrations were not statistically significant (*p* > 0.05). This suggests a potentially more subtle interaction between yeast strains and the quality of Premium wine [37]. However, Y1 also had the highest mannose concentration in this group of wines. In the comparison of both qualities, no significant differences were found (*p* < 0.05), except for in the case of Y3, which did differentiate between qualities, and where the Standard quality presented higher mannose values. In comparison with other studies, for example, that of Quirós et al. (2012) [38], significant differences were present between different yeast strains for the mannose content, with red wines reporting over 200 mg L^−1^ and from 100 to 150 mg L^−1^. Guaita et al. (2011) [39] reported a mannose content in wines of around 100 mg L^−1^. Usually, red wines have a content of 100 to 150 mg L^−1^ [40], so most of the strains used in this study can be considered overproducers.

In addition to mannose concentration, the phenolic composition of the wine matrix plays a critical role in modulating sensory attributes such as viscosity and mouthfeel. Phenolic compounds, including tannins and anthocyanins, are known to interact with mannoproteins, forming stable complexes that influence wine texture and reduce astringency [41]. Studies like that of Guadalupe et al. (2010) [18] have shown that wines with higher phenolic content demonstrate stronger interactions with polysaccharides, which can enhance viscosity and improve tactile sensations.

In this study, the differences between Standard and Premium wines may reflect variations in their phenolic composition, as Premium wines typically exhibit higher phenolic content due to superior ripening conditions. This matrix effect likely explains why viscosity correlations with mannose were less pronounced in Premium wines, where phenolics may have a greater influence on sensory perception. Future studies could benefit from including phenolic composition analysis to further elucidate the interplay between mannoproteins and phenolic compounds in determining wine sensory properties.

### 4.3. Rheological Behavior

The viscosity data revealed a consistent trend, with yeast strains significantly influencing the viscosity of both Standard- and Premium-quality wines. The flow behavior of the samples exhibited Newtonian characteristics in both Standard- and Premium-quality wines [15], as depicted in Figure 1. The robust fit of the linear model and high regression coefficients exceeding 0.95 in all instances validate the applicability of the Newtonian model to describe the flow properties. Newtonian behavior implies a constant viscosity under varying shear rates, providing a fundamental understanding of the flow dynamics of the wines [42]. Viscosity, a crucial parameter affecting wine mouthfeel and texture, is modulated by the mannoprotein-producing yeast strains [43]. The significant differences observed underscore the pivotal role of yeast selection in shaping the perceived viscosity of Cabernet Sauvignon wines.

In Standard-quality wines, Y1 stands out, with significantly higher viscosity compared to other yeast treatments (Figure 1A). This distinctive viscosity profile may be attributed to the higher level of mannoproteins produced by Y1, which results in increased resistance to flow [44]. The elevated viscosity in Standard-quality wines with Y1 suggests the potential for this yeast strain to impart a fuller and more substantial mouthfeel, contributing positively to the overall sensory experience [45]. In Premium-quality wines, distinctions in viscosity among samples were also evident, with Y3 displaying the highest viscosity (Figure 1B). The increased viscosity in Y3-treated wines could not be associated with mannoprotein concentration in Premium-quality Cabernet Sauvignon [46]. However, Y1 again in this case showed a high viscosity, not different from Y3. The distinct viscosity profiles between Standard- and Premium-quality wines highlight the intricate interplay between yeast characteristics and wine quality, emphasizing the potential for yeast selection to achieve specific mouthfeel goals.

As visualized, Standard-quality wines exhibited a moderate positive correlation (Pearson = 0.78; R^2^ = 0.60), and although it is not completely linear, it provides a good approximation for these wines. In contrast, for Premium wines, the correlation is very low and does not fit the model (Pearson = 0.30; R^2^ = 0.09). The observed variations in viscosity can be attributed to the mannoprotein content influenced by yeast strains. Mannoproteins, being large polysaccharide molecules, contribute to viscosity by influencing the overall structure and interactions within the wine matrix [47,48]. The increased viscosity in Y1-treated Standard-quality wines may have resulted from enhanced mannoprotein production or unique mannoprotein characteristics. Similarly, in the case of the elevated viscosity in Y3-treated Premium-quality wine, it could have been impacted by other factors, not only mannoproteins [49]. This is because viscosity in wines can also be influenced by the interactions between polysaccharides, ethanol content, phenolic compounds, and proteins, all of which contribute to the overall matrix and flow behavior of the wine [50]. For Premium wines, the nuanced structural complexity of these components, particularly in wines with higher-quality grape origins and extended aging, likely amplifies these effects.

The impact of yeast selection on viscosity, coupled with the observed differences between Standard- and Premium-quality wines, highlights the potential for tailored yeast choices to achieve specific mouthfeel attributes [51]. This understanding contributes to the broader goal of optimizing wine quality through informed yeast selection strategies, providing winemakers with a valuable tool for refining sensory characteristics in their final products [52].

Several studies have explored the role of mannoproteins in wine texture and sensory properties. For instance, Guadalupe et al. (2010) [18] demonstrated that yeast-derived mannoproteins stabilize color and influence mouthfeel characteristics, particularly in red wines. Their findings highlighted a positive relationship between mannoprotein content and wine viscosity, similar to what we observed in Standard-quality wines fermented with Y1. Likewise, Rinaldi et al. (2019) [9] reported that mannoproteins contribute to smoother textures and reduced astringency, which aligns with our sensory results for Premium-quality wines fermented with Y5.

However, unlike previous studies that primarily focused on commercial mannoprotein additives (e.g., that of Quirós et al., 2012 [37]), this study provides a comprehensive comparison of six yeast strains capable of overproducing mannoproteins directly during fermentation. This represents a practical and cost-effective approach for winemakers compared to external mannoprotein additions. Additionally, the nuanced differences observed between Standard- and Premium-quality wines regarding the correlation between mannoprotein concentration and viscosity have not been previously reported. Our results indicate that, while mannoproteins significantly influence viscosity and mouthfeel in Standard wines, their role is less pronounced in Premium wines, where polyphenolic content and ethanol appear to exert greater effects.

This study also stands out by integrating sensory analysis with rheological and compositional data, revealing distinct relationships between viscosity, mannoprotein content, and attributes like smoothness, roundness, and volume. These findings contribute novel insights into how yeast strain selection can be tailored to optimize sensory profiles in wines across different quality levels. Compared to earlier research, which often focused on individual parameters, our work presents a more holistic understanding of the interplay between mannoproteins, wine structure, and sensory perception.

### 4.4. Sensorial Attributes

According to the Rate-All-That-Apply (RATA) analysis presented in Table 5, the sensory attributes of Standard-quality Cabernet Sauvignon wines underwent significant alterations due to the different mannoprotein-producing yeasts. Interesting variations were observed in drying astringency, bitterness, structured, smoothness, and quality in mouth, underscoring the impact of yeast on these sensory characteristics [53]. In general, the Y1 yeast was the one that presented the highest values in all attributes, followed closely by the Y3 and Y5 yeasts.

Similar significant sensory differences were observed in Premium-quality Cabernet Sauvignon wines due to mannoprotein-producing yeasts (Table 6). In general, all treatments presented similar patterns, and the Y5 yeast could be distinguished in terms of parameters such as fat, velvety astringency, smoothness, and roundness, suggesting a positive impact on texture, balance, and overall quality [54]. These variations underscore the multifaceted role of yeast, which influences fermentation dynamics and contributes significantly to the perceived qualities of the final Premium wine product [55].

In Standard-quality wine, a discernible relationship group emerged, connecting astringency with quality in the mouth [56]. Simultaneously, another group, comprising structured, viscosity, round, fat, and mannose parameters, demonstrated a somewhat correlated relationship with volume [57]. Wines with Y4 and Y5 are predominantly found in the first group, whereas Y1 and Y3 are associated with the second group. Notably, Y2 and Y6 exhibited no discernible relationships with these parameters.

Conversely, within Premium-quality wine, two distinctive groups surfaced: one characterized by volume, mannose, and viscosity, and another featuring astringency, overall quality, smoothness, roundness, and fat. The former comprises wines with Y3, Y2, and Y1, while the latter primarily includes wine with Y5. In both PCAs, a consistent observation was the association between volume and viscosity, with the latter exhibiting a connection to mannose concentration [58].

Mannoproteins significantly enhance wine sensory attributes, particularly smoothness and roundness, through their interaction with the wine matrix. This study highlights the differences in how mannoprotein-producing yeast strains influence sensory profiles in Standard- and Premium-quality wines. While mannose concentration strongly correlates with smoothness in Standard wines, Premium wines exhibit a more nuanced interplay involving mannoproteins, polyphenols, and ethanol [40]. These findings emphasize the potential of tailored yeast selection to modulate sensory attributes effectively, providing a foundation for future research into optimizing yeast-driven sensory outcomes in wines.

Table 7 elucidates the findings from a Principal Component Analysis (PCA) conducted on sensory attributes, mannose concentration, and viscosity in Cabernet Sauvignon wines. Eigenvalues depict the variance captured by each principal component (PC), while the cumulative percentage of variability signifies the proportion of total variance explained by the PCs. For Standard-quality wines, the first PC (F1) holds a substantial eigenvalue (4.8), elucidating 43.3% of the variance, with the first three PCs cumulatively explaining 84.4%. In Premium-quality wines, F1 boasts a higher eigenvalue (5.4), explaining 48.7% of the variance, and the first three PCs cumulatively explain 90.2%. Correlation coefficients delineate the strength and direction of relationships between original variables and PCs. In Standard-quality wines, mannose (mg L^−1^) and viscosity (Pa·s) exhibit strong positive correlations with F1, signifying their significant contributions. In Premium-quality wines, mannose strongly correlates with F1, while viscosity spans F1 and F2. Factor scores convey each yeast strain’s contribution to each PC, with Y1 notably impacting F1 in Standard-quality wines, influencing mannose and viscosity. For Premium-quality wines, Y5 predominantly contributed to F1, emphasizing its role in shaping overall quality. Contributions (%) indicate the proportion of each yeast strain’s impact on total variance within each PC. In Standard-quality wines, Y1, Y2, and Y3 significantly impacted F1, F2, and F3, respectively. In Premium-quality wines, Y5 dominated F1, showcasing its substantial influence. F1 strongly associated mannose concentration, viscosity, and sensory attributes, illustrating yeast strains’ comprehensive impact on wine composition and quality [59]. Premium wines are characterized by a pronounced influence of mannose and viscosity (captured by F1). Specific yeast strains, particularly Y1 and Y5, play crucial roles in determining Cabernet Sauvignon wine quality [60]. The Y1 strain demonstrated a significant impact on viscosity and sensory attributes in Standard-quality wines, particularly roundness and structure. This aligns with its higher mannose concentration, suggesting its potential for enhancing mouthfeel. Meanwhile, Y3 exhibited a notable influence on viscosity and smoothness in Premium-quality wines, despite its lower mannose concentration. This highlights the complex interplay between mannoproteins and other structural components like phenolics in high-quality wines.

Beyond its scientific relevance, the method demonstrated in this study holds significant potential for commercialization. By leveraging mannoprotein-producing yeast strains, winemakers can fine-tune sensory attributes such as viscosity, smoothness, and roundness, catering to evolving consumer preferences. This approach not only offers cost-effective alternatives to commercial mannoprotein additives but also aligns with sustainable winemaking practices. Furthermore, its application extends beyond wine, potentially benefiting the production of other fermented beverages such as beer and cider, thus broadening its market impact.

## 5. Conclusions

This study provides novel insights into the role of mannoprotein-producing yeasts in modulating wine viscosity and mouthfeel. Unlike previous research that focused primarily on commercial mannoprotein additives, our work explores yeast-strain-specific contributions during fermentation. Strains such as Y1 in Standard-quality wines and Y3 in Premium-quality wines exhibited differentiated effects on viscosity, roundness, and smoothness, highlighting the potential for tailored yeast selection as a practical, sustainable, and cost-effective approach to enhance wine sensory properties across different quality levels.

Future research should aim to further elucidate the interactions of mannoproteins with other wine components, such as tannins, anthocyanins, and volatile compounds, which may have additional influences on sensory attributes. Moreover, exploring longer aging periods on lees could offer valuable insights into enhancing wine stability and mouthfeel. Investigating other yeast strains or combinations may provide further opportunities to optimize wine production across various quality categories. Finally, integrating consumer preference studies with sensory and chemical data would bridge the gap between scientific findings and practical applications, strengthening their impact on the wine industry.

## Figures and Tables

**Table 1:** Chemical composition of Standard-quality Cabernet Sauvignon wines fermented with different mannoprotein-producing yeasts.

Yeast	Alcohol (*v*/*v*)	Titratable Acid (g L^−1^) ^†^	Acetic Acid (g L^−1^)	pH	Glucose + Fructose (g L^−1^)	Color Intensity	Hue
Y1	12.25 ± 0.07 ^a^	3.70 ± 0.02 ^c^	0.34 ± 0.01 ^a^	3.50 ± 0.01 ^a^	1.63 ± 0.02 ^ab^	4.20 ± 0.99 ^a^	0.65 ± 0.01 ^a^
Y2	12.05 ± 0.07 ^a^	3.99 ± 0.03 ^a^	0.29 ± 0.01 ^b^	3.49 ± 0.01 ^ab^	1.63 ± 0.01 ^ab^	4.05 ± 0.92 ^a^	0.61 ± 0.02 ^a^
Y3	12.05 ± 0.07 ^a^	3.69 ± 0.06 ^c^	0.34 ± 0.01 ^a^	3.47 ± 0.01 ^b^	1.61 ± 0.01 ^ab^	4.30 ± 1.13 ^a^	0.61 ± 0.01 ^a^
Y4	12.15 ± 0.07 ^a^	3.84 ± 0.04 ^b^	0.33 ± 0.01 ^a^	3.51 ± 0.01 ^a^	1.65 ± 0.01 ^a^	6.20 ± 2.26 ^a^	0.63 ± 0.04 ^a^
Y5	12.30 ± 0.14 ^a^	3.74 ± 0.02 ^bc^	0.29 ± 0.01 ^b^	3.50 ± 0.01 ^a^	1.65 ± 0.01 ^a^	5.05 ± 0.78 ^a^	0.57 ± 0.01 ^a^
Y6	12.15 ± 0.21 ^a^	3.77 ± 0.01 ^bc^	0.26 ± 0.01 ^b^	3.50 ± 0.01 ^a^	1.58 ± 0.01 ^b^	4.60 ± 0.01 ^a^	0.59 ± 0.01 ^a^
*p*-value *	**0.3235**	**0.0007**	**0.0003**	**0.0142**	**0.0133**	**0.5501**	**0.0554**

* Data expressed as mean ± standard deviation, obtained from three repetitions for each yeast treatment, compared by ANOVA (*p* < 0.05). Different letters indicate significant differences at *p* < 0.05, obtained by Tukey’s multiple comparisons test. ^†^ Grams by liter of tartaric acid.

**Table 2:** Chemical composition of Premium-quality Cabernet Sauvignon wines fermented with different mannoprotein-producing yeasts.

Yeast	Alcohol (*v*/*v*)	Titratable Acid (g L^−1^) ^†^	Acetic Acid (g L^−1^)	pH	Glucose + Fructose (g L^−1^)	Color Intensity	Hue
Y1	14.10 ± 0.01 ^a^	3.95 ± 0.08 ^a^	0.36 ± 0.01 ^a^	3.51 ± 0.02 ^a^	1.67 ± 0.01 ^a^	10.85 ± 0.78 ^ab^	0.61 ± 0.01 ^a^
Y2	14.00 ± 0.14 ^a^	3.89 ± 0.12 ^ab^	0.22 ± 0.01 ^d^	3.51 ± 0.01 ^a^	1.67 ± 0.01 ^a^	11.85 ± 1.63 ^a^	0.60 ± 0.02 ^a^
Y3	14.05 ± 0.49 ^a^	3.61 ± 0.10 ^b^	0.36 ± 0.01 ^ab^	3.49 ± 0.01 ^a^	1.69 ± 0.03 ^a^	9.00 ± 1.13 ^ab^	0.58 ± 0.02 ^a^
Y4	13.95 ± 0.07 ^a^	3.70 ± 0.07 ^ab^	0.29 ± 0.02 ^bcd^	3.49 ± 0.02 ^a^	1.68 ± 0.01 ^a^	8.00 ± 0.42 ^b^	0.63 ± 0.07 ^a^
Y5	14.20 ± 0.28 ^a^	3.67 ± 0.05 ^ab^	0.29 ± 0.03 ^abc^	3.52 ± 0.02 ^a^	1.68 ± 0.01 ^a^	9.80 ± 0.57 ^ab^	0.62 ± 0.02 ^a^
Y6	13.85 ± 0.35 ^a^	3.69 ± 0.02 ^ab^	0.26 ± 0.02 ^cd^	3.49 ± 0.01 ^a^	1.68 ± 0.01 ^a^	8.50 ± 0.42 ^ab^	0.60 ± 0.03 ^a^
*p*-value *	**0.8509**	**0.0292**	**0.0013**	**0.5128**	**0.8214**	**0.0383**	**0.7476**

* Data expressed as mean ± standard deviation, obtained from three repetitions for each yeast treatment, compared by ANOVA (*p* < 0.05). Different letters indicate significant differences at *p* < 0.05, obtained by Tukey’s multiple comparisons test. ^†^ Grams by liter of tartaric acid.

**Table 3:** Effect of mannoprotein-producing yeast on the mannose concentration of Cabernet Sauvignon wines of Standard and Premium qualities.

Yeast (Y)	Mannose Concentration (mg L^−1^)		*p*-Value *
	Standard	Premium	
Y1	206 ± 12 ^aA^	180 ± 5 ^aA^	**0.1077**
Y2	166 ± 10 ^abA^	159 ± 1 ^aA^	**0.4738**
Y3	197 ± 8 ^abA^	167 ± 1 ^aB^	**0.0331**
Y4	170 ± 21 ^abA^	167 ± 11 ^aA^	**0.8830**
Y5	167 ± 15 ^abA^	169 ± 2 ^aA^	**0.9027**
Y6	145 ± 12 ^bA^	154 ± 12 ^aA^	**0.5360**
*p*-value *	**0.0341**	**0.0803**	

* Data expressed as mean ± standard deviation, obtained from three repetitions for each yeast treatment, compared by ANOVA (*p* < 0.05). Different lowercase letters indicate significant differences between yeasts in each column at *p* < 0.05, obtained by Tukey’s multiple comparisons. Different uppercase letters indicate significant differences between wine quality in each row at *p* < 0.05, obtained by Tukey’s multiple comparisons test.

**Table 4:** Effect of mannoprotein-producing yeast and quality on the viscosity of Cabernet Sauvignon wines.

Yeast	Wine Quality				*p*-Value *
	Standard		Premium		
	Viscosity (Pa·s) (*×*10^3^)	R^2^ **	Viscosity (Pa·s) (*×*10^3^)	R^2^ **	
Y1	1.60 ± 0.01 ^aA^	0.970	1.95 ± 0.01 ^abA^	0.964	**0.0887**
Y2	1.15 ± 0.01 ^bB^	0.995	1.85 ± 0.01 ^bcA^	0.949	**0.0101**
Y3	1.20 ± 0.01 ^bB^	0.980	2.15 ± 0.01 ^aA^	0.954	**0.0028**
Y4	1.25± 0.01 ^bB^	0.976	1.70 ± 0.01 ^cdA^	0.954	**0.0121**
Y5	1.20 ± 0.01 ^bB^	0.956	1.45 ± 0.01 ^eA^	0.967	**0.0377**
Y6	1.10 ± 0.01 ^bB^	0.985	1.60 ± 0.01 ^deA^	0.983	**0.0194**
*p*-value *	**0.0037**	**-**	**0.0002**	**-**	

* Data expressed as mean ± standard deviation, obtained from three repetitions for each yeast treatment in both wine qualities, compared by ANOVA (*p* < 0.05). Different lowercase letters indicate significant differences between yeasts in each column at *p* < 0.05, obtained by Tukey’s multiple comparisons test. Different uppercase letters indicate significant differences between wine quality in each row at *p* < 0.05, obtained by Tukey’s multiple comparisons test. ** R^2^ is the coefficient of determination according to Equation (1).

**Table 5:** Effect of mannoprotein-producing yeast on sensorial attributes, obtained by RATA analysis, of Standard-quality Cabernet Sauvignon wines.

Yeast	Attribute								
	Volume	Fat	Velvety Astringency	Drying Astringency	Bitter	Smoothness	Structured	Round	Quality in Mouth
Y1	1.83 ^a^	1.63 ^a^	0.65 ^a^	2.19 ^a^	1.46 ^a^	1.75 ^a^	1.08 ^a^	0.92 ^a^	4.02 ^ab^
Y2	1.38 ^a^	1.35 ^a^	0.58 ^a^	1.25 ^b^	0.81 ^ab^	1.71 ^a^	0.52 ^b^	0.50 ^a^	3.60 ^b^
Y3	1.60 ^a^	1.79 ^a^	0.31 ^a^	1.13 ^b^	0.94 ^ab^	1.58 ^a^	1.08 ^a^	1.02 ^a^	4.02 ^ab^
Y4	1.79 ^a^	1.63 ^a^	0.63 ^a^	2.54 ^a^	0.85 ^ab^	0.71 ^b^	0.94 ^a^	0.79 ^a^	4.31 ^ab^
Y5	1.63 ^a^	1.54 ^a^	0.81 ^a^	1.83 ^ab^	0.63 ^b^	1.50 ^a^	0.96 ^a^	0.65 ^a^	4.85 ^a^
Y6	1.67 ^a^	0.83 ^a^	0.48 ^a^	2.29 ^a^	0.90 ^ab^	1.25 ^ab^	0.54 ^b^	0.63 ^a^	3.98 ^ab^
*p*-value *	**0.0800**	**0.0790**	**0.1394**	**0.0370**	**0.0389**	**0.0066**	**0.0271**	**0.0948**	**0.0490**

* Data expressed as mean from panelist repetitions for each yeast fermentation in Standard wine quality, compared by ANOVA (*p* < 0.05). Different letters indicate significant differences at *p* < 0.05, obtained by Tukey’s multiple comparisons test. *n* panelists = 24.

**Table 6:** Effect of mannoprotein-producing yeast on sensorial attributes, obtained by RATA analysis, of Premium-quality Cabernet Sauvignon wines.

Yeast	Attribute								
	Volume	Fat	Velvety Astringency	Drying Astringency	Bitter	Smoothness	Structured	Round	Quality in Mouth
Y1P	1.33 ^ab^	1.33 ^bcd^	0.48 ^c^	1.55 ^a^	0.75 ^a^	1.60 ^ab^	0.83 ^a^	0.65 ^b^	4.55 ^a^
Y2P	1.58 ^ab^	1.75 ^ab^	0.38 ^cd^	1.80 ^a^	0.48 ^a^	1.45 ^b^	1.10 ^a^	0.75 ^b^	4.98 ^a^
Y3P	1.93 ^a^	1.30 ^cd^	0.73 ^b^	1.53 ^a^	0.48 ^a^	1.70 ^ab^	0.95 ^a^	0.83 ^b^	4.75 ^a^
Y4P	1.20 ^ab^	1.08 ^d^	0.38 ^cd^	1.88 ^a^	0.98 ^a^	1.73 ^ab^	1.20 ^a^	0.83 ^b^	4.63 ^a^
Y5P	1.38 ^ab^	1.98 ^a^	0.98 ^a^	1.93 ^a^	0.78 ^a^	2.08 ^a^	1.30 ^a^	1.33 ^a^	5.50 ^a^
Y6P	1.05 ^b^	1.58 ^abc^	0.28 ^d^	1.68 ^a^	0.95 ^a^	1.50 ^ab^	0.95 ^a^	0.73 ^b^	4.55 ^a^
*p*-value *	**0.0331**	**0.0013**	**0.0001**	**0.4497**	**0.0940**	**0.0467**	**0.3364**	**0.0038**	**0.1437**

* Data expressed as mean from panelist repetitions for each yeast fermentation in Premium wine quality, compared by ANOVA (*p* < 0.05). The same letters indicate no significant differences. *n* panelists = 20.

**Table 7:** Relationships from the Principal Component Analysis between the sensory data, mannose concentration, and viscosity of Standard- and Premium-quality red wine samples with mannoprotein-producing yeast.

	Standard Quality					Premium Quality				
	F1	F2	F3	F4	F5	F1	F2	F3	F4	F5
Eigenvalue	4.8	2.8	1.7	1.3	0.4	5.4	3.0	1.6	0.7	0.4
Variability (%)	43.3	25.3	15.7	12.1	3.5	48.7	27.4	14.1	6.4	3.4
Cumulative %	43.3	68.7	84.4	96.5	100.0	48.7	76.1	90.2	96.6	100.0
**Correlations**										
Mannose (mg L^−1^)	0.921	−0.365	−0.084	0.074	0.078	−0.058	0.432	−0.795	0.110	0.408
Viscosity (Pa s)	0.857	0.070	0.282	0.415	0.090	−0.736	0.650	−0.018	−0.189	0.008
Volume	0.679	0.645	0.293	−0.147	−0.127	−0.099	0.933	0.215	−0.254	−0.098
Fat	0.738	−0.164	−0.556	−0.068	0.338	0.672	0.097	0.525	0.497	0.129
Velvety Astringency	−0.021	0.598	−0.282	0.737	0.138	0.689	0.644	−0.256	0.130	−0.169
Drying Astringency	0.118	0.889	0.442	−0.006	0.037	0.799	−0.412	0.124	−0.347	0.239
Bitter	0.721	−0.164	0.647	0.184	−0.017	0.140	−0.851	−0.475	0.062	−0.165
Smoothness	0.162	−0.733	−0.037	0.574	−0.326	0.824	0.240	−0.484	−0.020	−0.169
Structured	0.908	0.113	−0.376	−0.118	−0.083	0.877	−0.152	0.085	−0.443	0.058
Round	0.874	−0.129	−0.026	−0.446	−0.140	0.966	0.180	−0.106	0.028	−0.148
Quality in Mouth	0.169	0.684	−0.638	0.076	−0.302	0.914	0.325	0.197	0.072	0.124
**Factor Scores**										
Y1	3.577	−0.072	1.355	1.282	−0.075	−2.258	0.475	−1.511	0.904	0.708
Y2	−2.541	−2.080	0.079	0.968	0.735	−0.257	0.396	2.218	−0.362	0.769
Y3	1.567	−2.211	−0.928	−1.624	−0.312	−1.409	2.838	−0.002	−0.409	−0.803
Y4	0.467	2.264	−0.059	−1.106	0.924	0.095	−1.815	−1.139	−1.466	0.056
Y5	−0.544	1.354	−2.167	1.084	−0.513	4.885	0.563	−0.383	0.492	−0.019
Y6	−2.526	0.745	1.719	−0.603	−0.758	−1.056	−2.457	0.817	0.841	−0.712
**Contributions (%)**										
Y1	44.7	0.0	17.7	20.6	0.2	15.9	1.2	24.5	19.4	22.3
Y2	22.6	25.8	0.1	11.7	23.1	0.2	0.9	52.8	3.1	26.3
Y3	8.6	29.2	8.3	33.1	4.2	6.2	44.5	0.0	4.0	28.7
Y4	0.8	30.6	0.0	15.3	36.6	0.0	18.2	13.9	51.0	0.1
Y5	1.0	11.0	45.3	14.7	11.3	74.3	1.8	1.6	5.7	0.0
Y6	22.3	3.3	28.5	4.6	24.6	3.5	33.4	7.2	16.8	22.5

## Data Availability

The data presented in this study are available upon request from the corresponding author. The data are not publicly available due to privacy restrictions from the FOVI 230072 project.
